# Precision and Error Propagation in Static MEMS-IMU Inertial Navigation: A Stochastic Time-Series Analysis

**DOI:** 10.3390/s26154685

**Published:** 2026-07-23

**Authors:** Mohammad Mahdi Kariminejad, Mohammad Ali Sharifi, Mir Abolfazl Mostafavi, Alireza Amiri-Simkooei

**Affiliations:** 1 School of Surveying and Geospatial Engineering, College of Engineering, University of Tehran, Tehran 1439957131, Iran; m.kariminejad@ut.ac.ir (M.M.K.); sharifi@ut.ac.ir (M.A.S.); 2 Department of Geomatic Sciences, Faculty of Forestry, Geography, and Geomatics, Université Laval, Quebec City, QC G1V 0A6, Canada; 3 Department of Control and Operations, Faculty of Aerospace Engineering, Delft University of Technology, 2629 HS Delft, The Netherlands; a.amirisimkooei@tudelft.nl

**Keywords:** MEMS-IMU, inertial navigation, stochastic modeling, error propagation, precision analysis, multivariate NNLS-VCE

## Abstract

This paper investigates the precision and stochastic error propagation of navigation solutions obtained from a low-cost microelectromechanical system inertial measurement unit (MEMS-IMU) under static conditions. A modern smartphone equipped with an MEMS-IMU was rigidly mounted at a calibrated fixed location to establish a zero-reference scenario, and inertial measurements were collected while the device remained stationary. The dataset was divided into 75 non-overlapping segments, each comprising 30 s of data sampled at 10 Hz, to enable statistically robust analysis. For each segment, velocity and position, which are theoretically zero under static conditions, were computed using strapdown inertial mechanization. A comprehensive statistical framework was then applied to characterize the stochastic behavior of both the raw inertial measurements and the derived navigation states. The methodology first assessed data normality, stationarity using the Augmented Dickey–Fuller (ADF) test, and variance homogeneity using Bartlett’s test. Subsequently, ARIMA models were identified and validated using the Ljung–Box (LB) test, while power spectral density (PSD) analysis provided complementary frequency-domain characterization. In addition, a multivariate, non-negative least squares variance component estimation (NNLS-VCE) method was employed to jointly estimate the variance components of multiple navigation state variables. The results demonstrate that the accelerometer and gyroscope measurements along all three axes are well characterized as stationary white-noise processes, with standard deviations in the order of $10^{-2}\;m/s^{2}$ and $10^{-4}\;\mathrm{rad}/s$, respectively. The estimated velocity random walk (VRW) coefficients are $\left[0.197,0.201,0.160\right]\;m/s/\sqrt{h}$, while the corresponding angular random walk (ARW) coefficients are $\left[0.009,0.012,0.008\right]\;\mathrm{rad}/\sqrt{h}$. In contrast, the derived velocity and position estimates exhibit random walk behavior caused by error accumulation in the inertial mechanization process and are best represented by ARIMA(0,1,0) and ARIMA(0,2,0) models, respectively, consistent with the corresponding Allan variance analysis. After 30 s of static navigation, the average standard deviations of the ENU velocity estimates are $\sigma_{v}=\left[0.77,0.44,0.29\right]\;m/s$, while the corresponding position standard deviations are $\sigma_{p}=\left[1.30,0.69,0.46\right]\;m$. The proposed framework provides a comprehensive approach for the stochastic modeling, precision assessment, and error characterization of low-cost MEMS-IMU navigation systems.

## 1. Introduction

Inertial navigation is a core component of many modern positioning and motion-tracking systems. It operates by continuously integrating inertial measurements, specifically linear acceleration and angular velocity, over time to estimate position, velocity, and attitude [1,2]. This technique, known as inertial navigation or strapdown inertial navigation, does not require any external infrastructure, making it particularly valuable in environments where satellite or radio signals are blocked, unreliable, or unavailable. These characteristics have made inertial navigation systems (INSs) indispensable across a wide range of domains, including aerospace, defense, robotics, automotive systems, and more recently, mobile and wearable technologies [3,4].

Despite its theoretical strengths, the practical implementation of inertial navigation faces critical challenges, primarily due to the cumulative nature of integration errors. Even small biases or noise in sensor measurements can grow significantly over time, leading to substantial drift in the estimated navigation parameters. For this reason, inertial navigation is often combined with external aiding systems, such as Global Navigation Satellite Systems (GNSSs) [5,6,7,8], Ultra-Wideband (UWB) [9,10,11,12], vision systems [13,14], Bluetooth Low Energy (BLE) [15], or Wi-Fi-based localization [16,17], to improve long-term accuracy through sensor fusion techniques.

The emergence of microelectromechanical system (MEMS) technology has revolutionized the availability and cost-effectiveness of inertial sensors. MEMS-based inertial measurement units (IMUs) are now widely used in consumer-grade devices such as smartphones, tablets, wearable devices, and Internet-of-Things (IoT) platforms. These IMUs offer small size, low power consumption, and low production cost, making them attractive for ubiquitous navigation and motion-tracking applications. However, MEMS IMUs suffer from inherent limitations, including high noise density, temperature sensitivity, bias instability, and poor scale-factor repeatability. These limitations significantly reduce the reliability and accuracy of standalone inertial navigation solutions, particularly in long-duration or precision-critical tasks [18,19].

To address these limitations, extensive research has been conducted on error modeling, calibration, and filtering techniques. One important area of study is the stochastic characterization of MEMS IMU output signals [20,21]. Understanding the statistical properties of raw sensor data, such as whether it follows white noise, random walk, or more complex stochastic processes, can guide the design of more effective filtering and estimation methods, including Kalman filtering and machine learning-based approaches. Furthermore, understanding how these errors propagate into position, velocity, and attitude estimates is essential to performance assessment and system design. For example, Kariminejad et al. investigated the impact of measurement error propagation on distance estimation accuracy in low-cost BLE-based indoor positioning systems [22]. They also highlighted the importance of accurate mathematical modeling in geodetic applications [23].

This paper focuses on the analysis of MEMS-IMU behavior under static conditions using a smartphone as a low-cost test platform. A modern smartphone equipped with an MEMS-IMU was fixed at a known, calibrated location, representing a zero-motion scenario. Inertial data were collected over an extended period in static mode, under the assumption that any non-zero outputs in position, velocity, or attitude result solely from sensor noise and modeling errors. The time-series data were segmented into 75 smaller sequences to evaluate the short-term behavior and statistical consistency of the navigation outputs across multiple realizations.

By analyzing both the raw sensor measurements and the derived navigation parameters, we aim to characterize the noise and error propagation in the strapdown inertial navigation process. Following the assessment of normality and variance homogeneity, which are fundamental to the statistical characterization of the data, the raw acceleration and angular velocity measurements along the three orthogonal axes were found to be well modeled as white-noise processes. However, when these measurements are integrated using strapdown mechanization equations, the resulting position and velocity estimates exhibit behavior consistent with a random walk process. These findings corroborate previous research on the stochastic behavior of MEMS-based inertial sensors while providing additional empirical validation using real-world smartphone data collected in a controlled static environment [24,25,26].

This work contributes to ongoing efforts to enhance the understanding of MEMS-IMU limitations and stochastic error behavior, particularly in the context of low-cost navigation systems. Two complementary and well-established statistical and time-series analysis frameworks were employed, operating in the time and frequency domains. The time-domain analysis includes, first of all, checking the normal distribution and homogeneity of variances, following the Augmented Dickey–Fuller (ADF) test, Autoregressive Integrated Moving Average (ARIMA) model fitting, and the Ljung–Box (LB) test, while the frequency-domain analysis is based on power spectral density (PSD) estimation. These methods were applied to assess stationarity and to model the noise characteristics of both the raw sensor measurements and the derived navigation outputs. In the geodetic community, the least squares variance component estimation (LS-VCE) method is a well-established approach to estimating unknown variance components associated with different types of measurements [27]. The main methodological contribution of this paper is the integration of LS-VCE into MEMS-IMU stochastic analysis, providing a multivariate statistical framework that complements conventional Allan variance-based methods [28]. This technique enables the quantitative assessment of output precision, specifically the random noise characteristics of raw MEMS-IMU measurements as well as the navigation parameters derived from strapdown mechanization equations. To facilitate reliable variance estimation for short, segmented time-series data, a multivariate NNLS-VCE formulation was adopted, owing to its improved numerical stability and performance under such conditions. Compared with Allan variance, the multivariate NNLS-VCE method offers two key advantages. First, it allows all segmented time series to be analyzed simultaneously within a single estimation framework. Second, in addition to identifying the stochastic noise model, it provides quantitative estimates of the magnitude of each noise component through variance component estimation.

The remainder of this paper is organized as follows: Section 2 reviews the relevant literature on error characterization in MEMS-IMU-based inertial navigation systems. Section 3 presents the proposed methodology, beginning with the assessment of data normality and homogeneity of variance, followed by the application of the ADF, ARIMA, and LB tests in conjunction with PSD analysis. It then describes the strapdown inertial navigation mechanization and concludes with the multivariate NNLS-VCE method for stochastic error characterization and precision estimation of the derived navigation states, followed by a comparison with the Allan variance method. Section 4 describes the experimental setup and discusses the corresponding results. Finally, Section 5 summarizes the main findings and outlines directions for future research.

## 2. Literature Review

Inertial navigation systems provide estimates of position, velocity, and attitude by integrating data from inertial sensors, specifically accelerometers and gyroscopes. While high-end INSs employ navigation-grade sensors to achieve excellent precision, their cost, size, and power consumption limit their applicability in low-cost and embedded platforms. In contrast, MEMS-IMUs have gained widespread adoption due to their low cost, small size, and ease of integration into consumer-grade devices such as smartphones, wearables, and IoT-enabled systems. However, the performance of MEMS IMUs is significantly limited by various sources of error, including sensor noise, bias instability, temperature sensitivity, and scale-factor variation [29,30,31].

To overcome these limitations, considerable research has focused on error modeling, stochastic characterization, and multi-sensor fusion approaches [32,33]. One well-known approach is INS/GNSS integration, which mitigates drift through external positioning updates [34]. In addition, several studies have emphasized the critical role of stochastic modeling in low-cost INSs, particularly in GNSS-denied environments [35,36,37]. Traditional noise characterization techniques, such as Allan variance (AV), PSD analysis, and autocorrelation function (ACF) analysis, have been widely used to identify underlying noise processes in inertial sensor data [28,38,39].

Some studies have focused on advanced time-series segmentation and short-term stochastic modeling to more accurately characterize inertial sensor behavior. Zhao et al. [40] employed the Generalized Method of Wavelet Moments (GMWM) to evaluate the noise characteristics of low-cost inertial sensors, highlighting the limitations of conventional stochastic error identification techniques, such as Allan variance and PSD analysis, in distinguishing and isolating individual error components in the spectral domain. Similarly, Blum et al. [41] demonstrated that the noise inherent in real inertial sensor measurements is time-correlated, that is, colored, and must be represented as a combination of multiple stochastic processes to accurately describe its underlying dynamics.

Recent research has applied advanced filtering and machine learning techniques to improve the modeling and calibration of inertial sensors [42,43,44]. However, relatively few studies have proposed a quantitative, multivariate framework capable of jointly estimating noise levels across all sensor outputs, namely, the three accelerometer and three gyroscope axes. Most existing approaches rely on single-channel diagnostics, such as Allan variance or power spectral density analysis, and often require subjective interpretation or manual tuning. In contrast, Balamuta et al. [45] introduced a computationally efficient framework based on the GMWM for the automatic and statistically consistent identification of stochastic processes in inertial sensors, highlighting the potential for unified noise modeling. Nonetheless, limited attention has been devoted to the stochastic characterization of embedded MEMS-IMUs in smartphones, particularly under controlled static, zero-motion conditions. Suvorkin et al. [46] and Radi et al. [47] investigated the stochastic performance of low-cost and smartphone-grade MEMS sensors, demonstrating that while these devices exhibit significant temperature- and environment-dependent noise, comprehensive multivariate stochastic modeling and the propagation of such errors into navigation parameters remain open challenges.

The growing demand for accurate, low-cost navigation solutions in smartphones and IoT devices underscores the necessity of reliable stochastic error modeling for MEMS-based IMUs. Accurate characterization of noise properties in both the raw sensor measurements, namely, linear acceleration and angular velocity, and the derived navigation states, including position, velocity, and attitude, is essential to robust system design, effective error mitigation, and rigorous uncertainty quantification [48]. Silva et al. [49] presented a comprehensive error analysis of coarse-alignment formulations for stationary strapdown inertial navigation systems (SINSs). Such analyses are particularly important in standalone inertial navigation scenarios where GNSS signals may be unavailable, including indoor environments, urban canyons, and underground settings.

To address the gaps in existing work, this paper introduces a novel stochastic modeling approach based on multivariate NNLS-VCE. Unlike conventional methods, NNLS-VCE enables the quantitative estimation of noise levels for each component of the raw sensor measurements, as well as the derived navigation parameters by decomposing the observed variability into identifiable stochastic processes, such as white noise, bias instability, and random walk, while enforcing non-negativity constraints on the variance components. This approach improves objectivity and statistical reliability compared with heuristic or manually tuned methods. Moreover, the proposed framework builds upon previous applications of NNLS-VCE for the stochastic modeling of GNSS time series [50], extending its applicability to MEMS-IMU-based inertial navigation.

In this study, a modern smartphone was rigidly mounted at a calibrated zero-motion location, and a long-duration static dataset of MEMS-IMU measurements was acquired. The collected data were partitioned into 75 non-overlapping segments to capture statistical variability across multiple realizations. For each segment, velocity and position estimates were computed using strapdown inertial navigation mechanization. Subsequently, the two aforementioned statistical frameworks were employed to assess stationarity and identify the underlying stochastic noise models. In addition, the NNLS-VCE method was applied to both the raw inertial measurements and the derived navigation states to quantitatively characterize their stochastic properties. The results indicate that the raw inertial measurements are well modeled as white-noise processes, whereas the derived navigation states exhibit random walk behavior, thereby confirming theoretical expectations and demonstrating the effectiveness of the proposed NNLS-VCE framework for fine-grained stochastic characterization.

## 3. Methodology

This section is divided into three main subsections, namely, statistical validation and noise model identification, strapdown mechanization and INS solution, and finally, noise quantification using multivariate NNLS-VCE.

### 3.1. Statistical Tests for Stationarity Check and Noise Modeling

First, the normality of the entire inertial measurement time series was assessed using Q–Q plots and histograms overlaid with fitted Gaussian curves. Subsequently, the stationarity of each time-series segment was evaluated using the ADF test, followed by Bartlett’s test to verify the homogeneity of variance across all segmented observations. These preliminary statistical assessments establish the validity of applying stochastic time-series models to the data. Next, ARIMA models were identified to characterize the temporal stochastic behavior of the measurements, and their adequacy was assessed using the LB test applied to the model residuals. Finally, a sensitivity analysis was conducted for different segment lengths (e.g., 10, 30, and 90 s), and the corresponding white-noise parameters, including their standard deviations, were estimated for each segment. In parallel, PSD analysis was performed to provide a complementary frequency-domain characterization of the noise processes.

The ADF test determines whether a time series is stationary by testing for the presence of a unit root. Stationarity is a fundamental assumption in stochastic time-series analysis because many modeling techniques rely on the statistical properties of the process remaining invariant over time. In contrast, Bartlett’s test evaluates whether the segmented observations possess statistically homogeneous variances, thereby supporting the assumption that they represent multiple realizations of the same stochastic process.

To model the stochastic behavior of both stationary and non-stationary signals, the ARIMA framework was adopted. Unlike ARMA models, ARIMA incorporates the differencing parameter *d*, enabling non-stationary series to be transformed into stationary ones while preserving their temporal dependence. The optimal ARIMA(p,d,q) model was selected automatically using the Akaike Information Criterion (AIC) and the Bayesian Information Criterion (BIC), which balance model accuracy against complexity. The fitted models were subsequently validated using the LB test, which examines whether the residuals exhibit significant autocorrelation. Residuals that are statistically indistinguishable from white noise indicate that the temporal dependencies of the original series have been adequately captured by the selected ARIMA model.

Complementing the time-domain analysis, PSD estimation was employed to investigate the distribution of signal power over frequency. PSD provides an independent frequency-domain assessment of the underlying noise characteristics and serves as a cross-validation of the stochastic behavior identified through the ADF, ARIMA, and Ljung–Box analyses. Together, these complementary approaches provide a comprehensive framework for characterizing the stochastic properties of both stationary inertial measurements and the derived non-stationary navigation states.

#### 3.1.1. Validation of the Gaussian Noise Assumption

Many stochastic estimation techniques employed in inertial navigation systems assume that the underlying sensor noise can be adequately represented by a Gaussian random process. In particular, the Kalman filter and its variants model the process and measurement noises as zero-mean Gaussian random variables, enabling recursive minimum-variance state estimation and covariance propagation. Consequently, prior to stochastic noise modeling and variance component estimation, the Gaussianity of the inertial measurements is assessed. Let the inertial measurement sequence of a given accelerometer or gyroscope axis be represented by a following time-series:

(1) $$y={\left[y_{1},y_{2},\ldots ,y_{N}\right]}^{T},$$

where *N* denotes the total number of observations. The sample mean and sample standard deviation are computed as

(2) $$\hat{\mu }=\frac{1}{N}\sum_{i=1}^{N}y_{i},$$

and

(3) $$\hat{\sigma }=\sqrt{\frac{1}{N-1}\sum_{i=1}^{N}{\left(y_{i}-\hat{\mu }\right)}^{2}},$$

respectively. Under the Gaussian assumption, the probability density function (PDF) of the measurements is given by

(4) $$f\left(y\right)=\frac{1}{\sqrt{2\pi {\hat{\sigma }}^{2}}}\exp\left(-\frac{{\left(y-\hat{\mu }\right)}^{2}}{2{\hat{\sigma }}^{2}}\right).$$

The first stage of the assessment consists of comparing the empirical histogram of the measurements with the theoretical Gaussian PDF defined by Equation (4). A close agreement between the two distributions indicates that the dominant stochastic behavior of the measurements can be adequately approximated by a Gaussian model.

In addition, quantile–quantile (Q–Q) plots are employed to compare the empirical quantiles of the measurements with the theoretical quantiles of a Gaussian distribution. The theoretical Gaussian quantiles are computed as

(5) $$q_{i}^{\left(g\right)}=\hat{\mu }+\hat{\sigma }\Phi^{-1}\left(p_{i}\right),$$

where $\Phi^{-1}\left(\cdot \right)$ denotes the inverse cumulative distribution function (CDF) of the standard normal distribution and $p_{i}$ represents the cumulative probability associated with the *i*-th ordered sample. The empirical quantiles are denoted by

(6) $$q_{i}^{\left(e\right)}=Q_{e}\left(p_{i}\right),$$

where $Q_{e}\left(\cdot \right)$ represents the empirical quantile function. For a Gaussian-distributed measurement sequence, the relationship

(7) $$q_{i}^{\left(e\right)}\approx q_{i}^{\left(g\right)},\;i=1,\ldots ,N,$$

should hold, resulting in an approximately straight-line pattern in the Q–Q plot.

The validation of the Gaussian noise assumption is particularly important in the context of inertial navigation because the covariance propagation equations and Kalman filtering framework commonly adopted in INS applications are derived under Gaussian noise assumptions. Furthermore, the stochastic noise models identified in this study are subsequently used in the multivariate NNLS-VCE procedure and navigation error propagation analysis. Therefore, demonstrating that the inertial measurements exhibit approximately Gaussian behavior increases confidence in the suitability of the adopted stochastic modeling framework and the resulting uncertainty estimates.

Although practical MEMS inertial sensors may exhibit slight deviations from perfect Gaussianity due to quantization effects, environmental influences, and low-frequency drift phenomena, reasonable agreement between the empirical and theoretical distributions is generally considered sufficient for stochastic modeling and Kalman filter-based inertial navigation applications.

#### 3.1.2. Augmented Dickey–Fuller (ADF) Test

The ADF test is a widely used statistical procedure for assessing the stationarity of a time series [51,52,53]. It tests the null hypothesis that the series contains a unit root, implying non-stationarity, against the alternative hypothesis of stationarity. The general form of the ADF regression model is expressed as

(8) $$\Delta y_{t}=a_{0}+a_{1}t+\gamma y_{t-1}+\sum_{i=1}^{h}\delta_{i}\Delta y_{t-i}+\varrho_{t},$$

where $\Delta y_{t}$ is the first-differenced series, $a_{0}$ and $a_{1}$ denote the constant and deterministic trend, γ is the coefficient of the lagged level term, *h* is the number of lagged difference terms included to remove serial correlation in the residuals $\varrho_{t}$, and *t* represents the time index.

Under the null hypothesis $H_{0}:\gamma =0$, the series possesses a unit root and is therefore non-stationary. Rejection of $H_{0}$ in favor of the alternative $H_{1}:\gamma <0$ indicates that the series is stationary. In this study, the ADF test was applied to the raw inertial measurements to determine whether the accelerometer and gyroscope outputs exhibit stationarity, thereby validating their suitability for subsequent stochastic noise modeling.

#### 3.1.3. Assessment of Variance Homogeneity Across Segments

The ADF test employed in the previous subsection evaluates the stationarity of each individual time-series segment. Nevertheless, stationarity alone does not ensure that different segments have identical variance characteristics. Because the proposed NNLS-VCE framework treats the segmented observations as multiple realizations of the same stochastic process, it is essential to verify that their variances are statistically homogeneous. To this end, Bartlett’s test is employed to examine the null hypothesis that all segments share a common variance. Let the inertial measurement sequence be divided into *K* non-overlapping segments, where the *k*-th segment contains $n_{k}$ samples,

(9) $${\left[y_{k,1},y_{k,2},\ldots ,y_{k,n_{k}}\right]}^{T},\;k=1,\ldots ,K.$$

The sample variance of the *k*-th segment is computed as

(10) $$s_{k}^{2}=\frac{1}{n_{k}-1}\sum_{i=1}^{n_{k}}{\left(y_{k,i}-{\bar{y}}_{k}\right)}^{2},$$

where

(11) $${\bar{y}}_{k}=\frac{1}{n_{k}}\sum_{i=1}^{n_{k}}y_{k,i}$$

denotes the sample mean of the *k*-th segment. To assess variance homogeneity among all segments, Bartlett’s test is employed. The null hypothesis is defined as

(12) $$H_{0}:\;\sigma_{1}^{2}=\sigma_{2}^{2}=\ldots =\sigma_{K}^{2}$$

while the alternative hypothesis is

(13) $$H_{1}:\;\exists \;i,j\in \left\{1,\ldots ,K\right\},\;i\neq j,\;\mathrm{such}\mathrm{that}\;\sigma_{i}^{2}\neq \sigma_{j}^{2}$$

Bartlett’s test statistic is computed as

(14) $$\Pi =\frac{\left(N-K\right)\ln\;\left(s_{p}^{2}\right)-\sum_{k=1}^{K}\left(n_{k}-1\right)\ln\;\left(s_{k}^{2}\right)}{1+\frac{1}{3(K-1)}\left[\sum_{k=1}^{K}\frac{1}{n_{k}-1}-\frac{1}{N-K}\right]}$$

where

(15) $$N=\sum_{k=1}^{K}n_{k}$$

is the total number of samples and

(16) $$s_{p}^{2}=\frac{\sum_{k=1}^{K}\left(n_{k}-1\right)s_{k}^{2}}{N-K}$$

is the pooled variance estimate. Under the null hypothesis, the test statistic approximately follows a chi-square distribution:

(17) $$\Pi \overset{H_{0}}{∼}\chi_{K-1}^{2}$$

(18) $$p_{B}=P\left(\chi_{K-1}^{2}\geq \Pi \right)$$

A significance level of α=0.05 is adopted. If the resulting $p_{B}$-value exceeds the significance threshold, the null hypothesis cannot be rejected, indicating that the variances of the segmented measurements are statistically homogeneous.

The assessment of variance homogeneity is particularly important in the present study because the subsequent stochastic noise characterization and multivariate NNLS-VCE estimation are performed using all segments collectively. If the segment variances remain statistically consistent, the segmented observations can be regarded as repeated realizations of the same stochastic process, thereby supporting the validity of the estimated variance components and the resulting inertial navigation uncertainty analysis. Conversely, substantial variance differences among segments may indicate non-stationary behavior, environmental effects, or time-varying sensor characteristics that could bias the stochastic parameter estimation.

#### 3.1.4. ARIMA Model Fitting and Order Selection

After assessing normality, stationarity using the ADF test, and homogeneity of variances, an appropriate stochastic model is required to characterize the underlying noise processes [54]. The ARIMA model is adopted in this study because it provides a unified framework for modeling both the stationary behavior observed in the raw MEMS-IMU measurements and the non-stationary behavior exhibited by the derived inertial navigation states. In particular, random walk processes are naturally represented through the differencing parameter d, which transforms a non-stationary series into a stationary one while preserving its temporal dependence. In contrast, ARMA models are restricted to stationary time series because they do not incorporate a differencing operation.

An ARIMA(p,d,q) model is expressed as

(19) $$\Phi_{p}\left(B\right){\left(1-B\right)}^{d}y_{t}=\Theta_{q}\left(B\right)\varepsilon_{t},$$

where $y_{t}$ denotes the observed time series, *B* is the backward shift operator defined as $By_{t}=y_{t-1}$, $\varepsilon_{t}$ is a white-noise process, and (p,d,q) represent the autoregressive order, degree of differencing, and moving average order, respectively. The polynomials $\Phi_{p}\left(B\right)$ and $\Theta_{q}\left(B\right)$ are defined as

(20) $$\begin{array}{cc} \Phi_{p}\left(B\right) & =1-\phi_{1}B-\phi_{2}B^{2}-\ldots -\phi_{p}B^{p},\\ \Theta_{q}\left(B\right) & =1+\theta_{1}B+\theta_{2}B^{2}+\ldots +\theta_{q}B^{q}, \end{array}$$

In an ARIMA(p,d,q) model, the autoregressive (AR) coefficients $\phi_{1},\ldots ,\phi_{p}$ must satisfy the stationarity condition, meaning that all roots of the characteristic polynomial

(21) $$1-\phi_{1}z-\phi_{2}z^{2}-\ldots -\phi_{p}z^{p}=0$$

lie outside the unit circle, i.e., |z|>1.

Similarly, the moving average (MA) coefficients $\theta_{1},\ldots ,\theta_{q}$ must satisfy the invertibility condition, such that all roots of

(22) $$1+\theta_{1}z+\theta_{2}z^{2}+\ldots +\theta_{q}z^{q}=0$$

also lie outside the unit circle.

These conditions ensure that the ARIMA model is well defined, with a stationary behavior for the differenced series and a unique and stable representation for forecasting.

Fitting an ARIMA model involves (i) model identification and order selection and (ii) parameter estimation and model validation. For order selection, the AIC and BIC are used:

(23) $$\mathrm{AIC}=2\zeta -2\ln\left(\hat{L}\right),\;\mathrm{BIC}=\zeta \ln\left(\eta \right)-2\ln\left(\hat{L}\right),$$

where ζ denotes the number of estimated parameters, $\hat{L}$ is the maximized likelihood value, and η is the sample size. The ARIMA model with the lowest AIC or BIC value is selected as the preferred model.

After estimating the model parameters using maximum likelihood estimation (MLE), the residuals are evaluated with the LB test to ensure they behave as white noise, confirming that the temporal dependencies of the time series are properly captured. While ARMA models are suitable for stationary stochastic processes, they cannot directly handle non-stationary signals such as the random walk errors commonly observed in MEMS-IMU-derived velocity and position. In contrast, ARIMA models include a differencing step that allows for the accurate representation of non-stationary behaviors, making them the preferred choice for modeling integrated navigation errors. Table 1 summarizes the key differences between ARMA and ARIMA models, highlighting their applicability to MEMS-IMU stochastic error analysis.

#### 3.1.5. Ljung–Box (LB) Test for Residual Evaluation

Once an appropriate ARIMA model has been fitted to the time-series data, it is essential to verify that the model residuals behave as white noise; that is, they exhibit no significant autocorrelation over time. The LB test is a widely used statistical method for this purpose [55,56]. It provides a diagnostic measure of whether any remaining structure in the data has been adequately captured by the fitted model. The LB statistic Ψ is defined as

(24) $$\Psi =m\left(m+2\right)\sum_{\tau =1}^{h}\frac{{\hat{r}}_{\tau }^{2}}{m-\tau },$$

where *m* is the number of observations, *h* is the number of lags being tested, and ${\hat{r}}_{\tau }$ denotes the sample autocorrelation at lag τ computed from the model residuals. Under the null hypothesis of no autocorrelation, Ψ approximately follows a chi-square ($\chi^{2}$) distribution with (h−p−q) degrees of freedom, where (p,q) are the autoregressive and moving average orders of the fitted ARIMA model. The hypotheses of the LB test are formulated as:

(25) $$\begin{array}{cc} H_{0} & :\;\mathrm{Residuals}\;\mathrm{are}\;i.i.d.\;(\mathrm{white}\;\mathrm{noise}),\\ H_{1} & :\;\mathrm{Residuals}\;\mathrm{exhibit}\;\mathrm{significant}\;\mathrm{autocorrelation}. \end{array}$$

Under $H_{0}$, the residuals are assumed to contain no remaining temporal dependence, implying that the ARIMA model has successfully captured the underlying structure of the time series. Rejection of $H_{0}$ indicates that the residuals retain statistically significant correlations, suggesting that the model specification may be inadequate or require higher-order terms.

If the computed probability is below the chosen significance level (commonly α = 0.05), the null hypothesis is rejected, implying that the residuals still contain autocorrelation and that the model may require refinement. Conversely, if the computed probability exceeds the significance level, the null hypothesis cannot be rejected, indicating that the residuals are uncorrelated and that the fitted ARIMA model adequately represents the underlying stochastic process.

In this study, the LB test is applied as a post-modeling diagnostic to validate the ARIMA-based stochastic models of MEMS-IMU noise. Passing the LB test confirms that the residual sequence can be treated as white noise, ensuring that the modeled process captures the temporal dynamics of the raw inertial measurements effectively.

#### 3.1.6. Noise Model Identification Using the Slope of the PSD

The slope of the PSD on a log–log scale provides valuable information about the stochastic noise type underlying a time series [57,58]. This approach is rooted in power-law noise modeling, widely used in inertial sensor characterization [46], geodetic time-series analysis [59,60], and general stochastic process modeling. Many stochastic processes exhibit a power-law spectral form:

(26) $$S\left(\nu \right)\propto \nu^{\kappa },$$

where κ denotes the spectral slope. Taking the logarithm of both sides yields

(27) $$\log S\left(\nu \right)=\kappa \log\nu +C_{0},$$

indicating that in a log–log PSD plot, the spectrum appears as a straight line with slope κ. Different noise processes correspond to characteristic slope values [61], as summarized in Table 2.

The slope thus provides a direct method for identifying the governing noise model. The practical procedure for determining the noise type from the PSD is as follows:

1. Compute the PSD of the residual or time-series data.
2. Convert both frequency and PSD values to a logarithmic scale.
3. Fit a linear regression model of the form
  (28) $$\log S\left(\nu \right)=\kappa \log\nu +C_{0},$$
  where κ is the spectral slope and $C_{0}$ is a constant.
4. Compare the estimated slope κ with known spectral signatures (see Table 2) to identify the noise type.
5. Apply the inferred noise model in subsequent filtering, Kalman filtering, or residual diagnostic analysis.

### 3.2. Strapdown Mechanization and INS Solution

Strapdown and space-stabilized mechanization represent two fundamental approaches in the design of INSs. In strapdown systems, inertial sensors are rigidly mounted onto the body of the platform, and all orientation changes must be determined through computational algorithms. In contrast, space-stabilized systems utilize a gimballed platform that maintains a constant orientation relative to an inertial reference frame, independent of platform motion. While space-stabilized systems simplify the computational burden by mechanically preserving alignment with the navigation frame, they are generally larger, heavier, and more costly, which limits their applicability in compact or cost-sensitive platforms. Conversely, strapdown systems offer significant advantages in terms of reduced size, lower cost, improved reliability, and mechanical simplicity, but they necessitate more sophisticated and computationally intensive algorithms to accurately track orientation and navigation parameters. As a result, strapdown mechanization has become the preferred choice in modern applications, particularly where system miniaturization and integration with embedded computing platforms are critical. SINSs compute position, velocity, and attitude by numerically integrating acceleration and angular velocity measurements provided by an IMU. Unlike gimballed systems, in strapdown configurations, the sensors are fixed to the body of the platform; therefore, the navigation equations must account for changes in orientation in real time. The accuracy of strapdown mechanization is highly sensitive to sensor noise, biases, and integration errors, which makes this approach an ideal context for stochastic analysis and error modeling. A comprehensive understanding of the coordinate reference frames involved in these computations is essential to interpreting and implementing the inertial navigation equations correctly. In this context, four commonly used reference frames are defined as follows:

1. Body frame (b-frame): A coordinate system fixed to the sensor or vehicle, where the inertial measurements (accelerations and angular rates) are directly observed.
2. Navigation frame (n-frame): A local-level frame, typically defined as East–North–Up (ENU) or North–East–Down (NED), which serves as the operational reference for position and velocity estimation.
3. Earth-Centered Inertial (ECI) frame: A non-rotating frame with its origin at the Earth’s center, used for high-precision inertial computations in space applications.
4. Earth-Centered Earth-Fixed (ECEF) frame: A rotating Earth-fixed frame, also centered at the Earth’s center, commonly used for global geo-referencing and satellite navigation.

For short-term static or local experiments, such as those conducted in this study, the body and navigation frames are of primary importance, while the ECI and ECEF frames play a secondary role due to their negligible impact on local-level motion estimation. The inertial navigation solution, in this study, generally consists of three principal computational steps. These steps sequentially process the inertial sensor measurements to estimate the navigation states, position and velocity of the system.

In this research study, to enhance the understanding of navigation output accuracy, the results are presented in the navigation frame (ENU frame). Also, in high-precision or long-duration inertial navigation, especially when using an ECEF or ECI frame, Coriolis and centripetal accelerations must be considered. In local-level navigation (like ENU or NED frames) and especially in short-duration or static conditions (as in our case), they are often negligible and thus omitted for simplicity.

#### 3.2.1. Attitude Update

Let $\omega_{ib}^{b}$ be the angular velocity of the body frame with respect to the inertial frame (what gyros measure), expressed in the body frame. The attitude is represented by a direction cosine matrix (DCM) $C_{b}^{n}$, which transforms vectors from body to navigation frame. The differential equation governing the DCM is given by

(29) $${\dot{C}}_{b}^{n}=C_{b}^{n}{\left[\omega_{ib}^{b}\right]}_{\times },$$

where ${\left[\omega_{ib}^{b}\right]}_{\times }$ denotes the skew-symmetric matrix constructed from the gyroscope measurements:

(30) $${\left[\omega_{ib}^{b}\right]}_{\times }=\left[\begin{array}{ccc} 0 & -\omega_{z} & \omega_{y}\\ \omega_{z} & 0 & -\omega_{x}\\ -\omega_{y} & \omega_{x} & 0 \end{array}\right].$$

We assume that the angular velocity measurements remain constant over each sampling interval. Under this assumption, the analytical solution of the first-order differential equation can be expressed as

(31) $$C_{b}^{n}\left(t_{0}+\Delta t\right)=C_{b}^{n}\left(t_{0}\right)\exp\;\left({\left[\omega_{ib}^{b}\right]}_{\times }\;\Delta t\right),$$

where Δt denotes the sampling interval and exp(·) represents the matrix exponential.

By increasing the number of samples, the algorithm eventually reaches its accuracy limit. Beyond this point, adding more samples only amplifies numerical errors rather than improving the performance of the rotation-vector algorithm [62]. Alternatively, quaternions or Euler angles can be used for attitude representation, with quaternions often preferred due to their numerical stability [63].

#### 3.2.2. Velocity Update

The specific force measured by the accelerometer, $f^{b}$, must be transformed to the navigation frame and corrected for gravity to update the velocity:

(32) $$v^{n}\left(t+\Delta t\right)=v^{n}\left(t\right)+\left(C_{b}^{n}\;f^{b}+g^{n}\right)\Delta t,$$

where $v^{n}$ is the velocity in the navigation frame, $C_{b}^{n}\cdot f^{b}$ is the specific force in the navigation frame, and $g^{n}$ is the gravity vector (e.g., ${\left[0,0,9.81\right]}^{T}$ in the NED frame for static scenarios).

#### 3.2.3. Position Update

Position is obtained by integrating the velocity over time:

(33) $$x^{n}\left(t+\Delta t\right)=x^{n}\left(t\right)+v^{n}\left(t\right)\;\Delta t.$$

In local-level applications, this position is usually expressed in a local Cartesian system (e.g., ENU or NED) or approximated using a flat Earth model when the displacement is small—as in this static test setup.

#### 3.2.4. Strapdown Mechanization Workflow and Static-Mode Considerations

**Figure 1:**
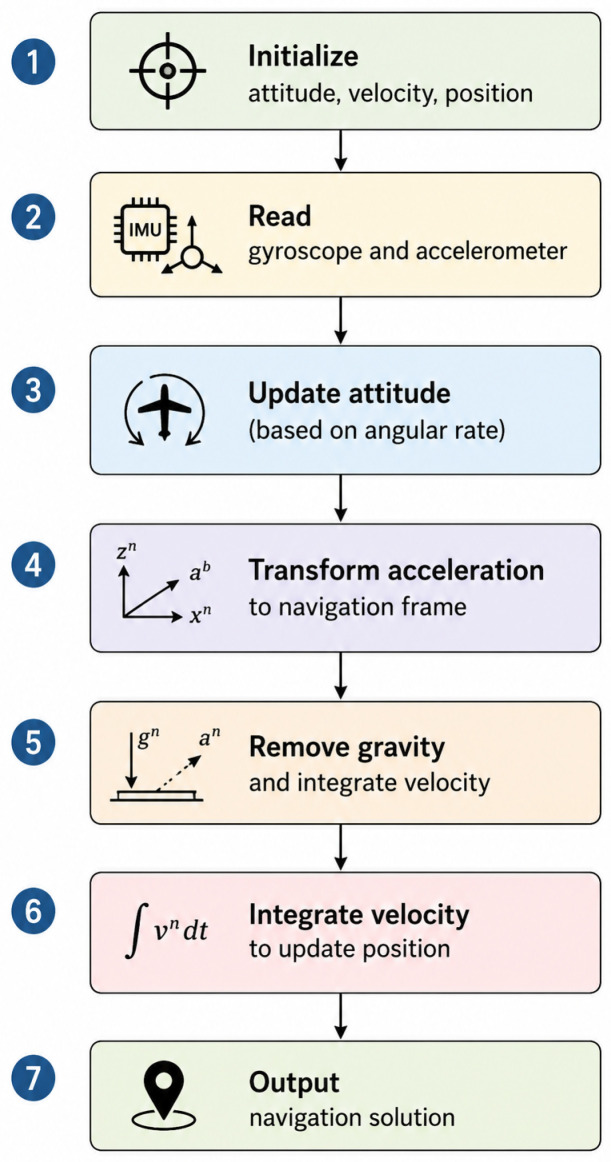
Simplified SINS mechanization flowchart.

Strapdown inertial navigation forms the core computational framework that transforms raw inertial measurements—namely, the angular rates from gyroscopes and the specific forces from accelerometers—into continuous estimates of attitude, velocity, and position. Because the inertial sensors are rigidly attached (“strapped down”) to the body of the platform, all measurements are inherently expressed in the body reference frame. The mechanization algorithm sequentially integrates these measurements to determine the navigation state, with the major processing stages conceptually illustrated in Figure 1.

In the context of this study, the IMUs are maintained in a static configuration, meaning that the true velocity and position are theoretically zero. Under such conditions, any non-zero estimates produced by strapdown mechanization arise solely from stochastic sensor noise, deterministic biases, scale-factor errors, and numerical integration effects. This makes the static environment a controlled and informative setting for examining how these error sources propagate through the mechanization chain.

Furthermore, static-mode analysis enables rigorous evaluation of stochastic noise models by contrasting the theoretically stationary navigation states with the observed behavior of the computed outputs. This comparison is particularly valuable for characterizing the performance of MEMS-based IMUs, whose accuracy and long-term stability are strongly influenced by the structure of their underlying noise processes. Such insights are essential to developing reliable stochastic models and improving navigation performance in applications where precision is critical.

### 3.3. Noise Quantification Using Multivariate NNLS-VCE

#### 3.3.1. Theory of NNLS-VCE

The LS-VCE method is a classical approach grounded in the principles of least squares estimation. Initially introduced by Teunissen (1988) and further refined by Teunissen and Amiri-Simkooei [27], LS-VCE has been widely applied to estimate variance components in stochastic models. However, a well-known limitation of LS-VCE is that it may yield negative variance estimates, which are physically and statistically inadmissible. To address this issue, Amiri-Simkooei [64] proposed the NNLS-VCE method. This technique reformulates the LS-VCE problem as a convex optimization problem subject to non-negativity constraints on the variance components. By incorporating the Karush–Kuhn–Tucker (KKT) optimality conditions into the solution framework, NNLS-VCE guarantees that all estimated variance components are non-negative. It should be noted that the reliability of the estimated variance components depends on the quality of the input data; therefore, outliers should be identified and removed prior to applying the NNLS-VCE method.

Given the convexity of the objective function, NNLS-VCE offers an efficient and computationally tractable solution, especially when compared with more general nonlinear optimization techniques. This makes the approach particularly well suited for large datasets or real-time processing scenarios.

In the context of GNSS and inertial sensor time-series analysis, NNLS-VCE can be effectively applied by augmenting the LS-VCE cost function F(σ) with the constraint σ≥0, thereby guaranteeing that all estimated variance components remain physically meaningful [64].

(34) $$\hat{\sigma }=\underset{\sigma \geq 0}{\arg\min}\;F\left(\sigma \right)=\underset{\sigma \geq 0}{\arg\min}\left(\frac{1}{2}\sigma^{T}N\sigma -l^{T}\sigma \right),$$

where N and l are defined as

(35) $$n_{kl}=\frac{1}{2}\mathrm{tr}\left(Q_{y}^{-1}P_{A}^{⊥}Q_{k}Q_{y}^{-1}P_{A}^{⊥}Q_{l}\right),$$

and

(36) $$l_{k}=\frac{1}{2}{\hat{e}}^{T}Q_{y}^{-1}Q_{k}Q_{y}^{-1}\hat{e},$$

where

(37) $$P_{A}^{⊥}=I-A{\left(A^{T}Q_{y}^{-1}A\right)}^{-1}A^{T}Q_{y}^{-1}$$

is the orthogonal projector.

#### 3.3.2. NNLS-VCE in Multivariate Models

To apply the method within a multivariate analysis framework, the complete static time series of measurements is segmented into shorter intervals of 30 s. For each segmented time series, the SINS solution is computed independently. This segmentation facilitates a more detailed and statistically reliable assessment of the stochastic characteristics of the derived navigation parameters. Based on this segmentation, the multivariate analysis leads to the following observation equation for each navigation component, i.e., ${\left[E,N,U,V_{E},V_{N},V_{U}\right]}^{T}$:

(38) $$E\left(\mathrm{vec}\left(Y\right)\right)=\left(I_{s}⊗A\right)\mathrm{vec}\left(X\right),$$

with covariance matrix

(39) D(vec(Y))=Σ⊗Q,

where Y is the matrix of separated computed parameters, e.g., $Y=[y_{1},y_{2},\ldots ,y_{s}]$; $I_{s}$ denotes the identity matrix of size *s*; and Σ represents the noise covariance matrix. For a comprehensive treatment of the properties of the vec-operator and the Kronecker product, readers are referred to Magnus (1988). For the multivariate linear model, we have [65]:

(40) $$n_{kl}=\frac{s}{2}\mathrm{tr}\left(Q^{-1}P_{A}^{⊥}Q_{k}Q^{-1}P_{A}^{⊥}Q_{l}\right),$$

**Figure 2:**
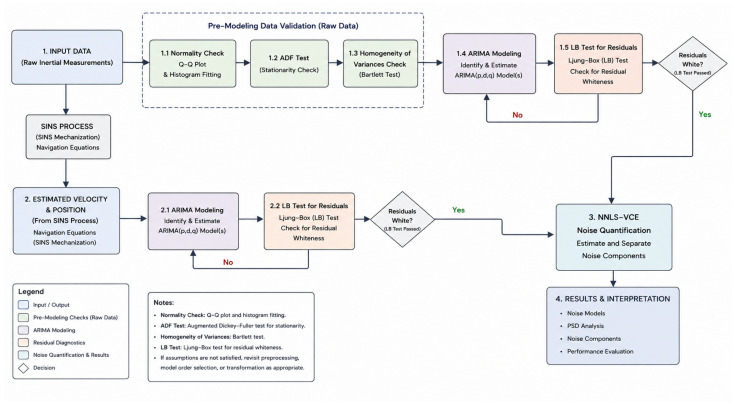
Methodology flowchart of the proposed approach.

If the covariance matrix Σ is unknown, a two-step procedure is employed to estimate the variance components. First, Σ is estimated, and then the preceding formulation is applied. This yields the vector $l={\left[l_{1},\ldots ,l_{s}\right]}^{T}$ with elements

(41) $$l_{k}=\frac{m-n}{2}\mathrm{tr}\left({\hat{E}}^{T}Q^{-1}Q_{k}Q^{-1}\hat{E}{\left({\hat{E}}^{T}Q^{-1}\hat{E}\right)}^{-1}\right),$$

where

(42) $$\hat{E}=P_{A}^{⊥}=\left[{\hat{e}}_{1},{\hat{e}}_{2},\ldots ,{\hat{e}}_{s}\right],$$

and ${\hat{e}}_{i}\in R^{m}$ denotes the least squares residual vector corresponding to the *i*-th time series. The NNLS-VCE method offers several advantages over the MLE method, which can be highlighted as follows. The NNLS-VCE method is relatively easy to comprehend and interpret compared with the MLE method. Estimates of negative variance components can result in a covariance matrix that is not positive definite. This means that the matrix does not have physically meaningful interpretations. Large negative variance components generally indicate potential flaws or issues in the stochastic model. NNLS-VCE is a straightforward method to implement it, which is also suitable for both univariate and multivariate analyses. It stands out for its practical efficiency, primarily due to its low computational burden. One advantage of NNLS-VCE is that it utilizes the Newton–Raphson method, which contrasts with the simplex procedure employed in MLE. The complete methodology is illustrated in Figure 2.

#### 3.3.3. Allan Variance-Based Noise Characterization and Comparison

To further validate the stochastic parameters obtained using the proposed multivariate NNLS-VCE approach, independent noise characterization was performed using Allan variance analysis. Allan variance is a widely adopted technique for the stochastic characterization of inertial sensors and has been extensively used to identify random noise processes in accelerometers and gyroscopes. In this study, Allan variance serves as a benchmark for evaluating the consistency of the variance components estimated by the proposed methodology. Let the inertial sensor measurements be represented by the discrete sequence

(43) $$y_{i},\;i=1,2,\ldots ,N,$$

sampled at interval

(44) $$T_{s}=\frac{1}{f_{s}},$$

where $f_{s}$ denotes the sampling frequency. For an averaging time

(45) $$\tau =mT_{s},$$

the averaged measurements are computed as

(46) $${\bar{y}}_{k}\left(\tau \right)=\frac{1}{m}\sum_{i=(k-1)m+1}^{km}y_{i},\;k=1,\ldots ,M$$

where *m* denotes the number of samples within each averaging interval. The Allan variance is then defined as

(47) $$\sigma_{A}^{2}\left(\tau \right)=\frac{1}{2(M-1)}\sum_{k=1}^{M-1}{\left({\bar{y}}_{k+1}\left(\tau \right)-{\bar{y}}_{k}\left(\tau \right)\right)}^{2}$$

where *M* denotes the total number of averaging intervals. The Allan deviation is obtained as

(48) $$\sigma_{A}\left(\tau \right)=\sqrt{\sigma_{A}^{2}\left(\tau \right)}$$

The Allan deviation is typically represented on a log–log scale as a function of the averaging time τ. Different stochastic processes produce characteristic slopes that enable identification of the dominant noise mechanisms. In particular,

(49) $$\sigma_{A}\left(\tau \right)\propto \tau^{-1/2}$$

indicates white noise, whereas

(50) $$\sigma_{A}\left(\tau \right)\propto \tau^{+1/2}$$

indicates a random walk process. For gyroscope measurements, the angle random walk (ARW) coefficient is obtained from the white-noise region of the Allan deviation curve as

(51) $$\mathrm{ARW}=\sigma_{A}\left(\tau \right)\sqrt{\tau }{|}_{\tau =\tau_{w}}$$

where ARW is typically expressed in units of $\deg/\sqrt{h}$ or $\mathrm{rad}/\sqrt{s}$. Similarly, for accelerometer measurements, the velocity random walk (VRW) coefficient is computed as

(52) $$\mathrm{VRW}=\sigma_{A}\left(\tau \right)\sqrt{\tau }{|}_{\tau =\tau_{w}}$$

where VRW is typically expressed in units of $m/s/\sqrt{h}$ or $m/s/\sqrt{s}$. Based on the Allan deviation analysis, only the white-noise region corresponding to ARW for the gyroscope and VRW for the accelerometer could be reliably identified. Although Bias Instability (BI) and Rate Random Walk (RRW) are common stochastic error processes in inertial sensors, no distinct zero-slope (BI) or +1/2-slope (RRW) regions were observed in the Allan deviation curves. This is primarily attributed to the relatively short data acquisition period (40 min at 10 Hz) and the characteristics of the smartphone-grade MEMS inertial sensors. In addition, the gradual increase in the device temperature due to battery and processor heating during data collection may introduce deterministic temperature-dependent bias drift, which can mask the stochastic bias instability and prevent the formation of a well-defined BI plateau. Consequently, BI and RRW were not estimated in this study, and only the ARW and VRW parameters were considered for stochastic noise characterization.

It should be noted that Allan variance and NNLS-VCE pursue similar objectives but rely on different estimation principles. Allan variance identifies stochastic processes through the scaling behavior of the signal over multiple averaging intervals, whereas NNLS-VCE directly estimates variance components by solving a constrained variance component estimation problem. Therefore, the comparison presented herein serves as an independent validation of the proposed stochastic characterization framework.

## 4. Field Experiment and Results

A comprehensive case study was conducted to characterize the random errors inherent in MEMS-based inertial navigation sensors operating under static conditions. For this purpose, inertial data comprising specific force (acceleration) and angular velocity measurements were collected from a Samsung Galaxy S22+ smartphone over an observation period of approximately 40 min within a calibrated laboratory environment. During the experiment, the smartphone was maintained in a fixed position, and its true location was assumed to be $x={\left[0,0,0\right]}^{T}$. Consequently, under ideal static conditions, the true values of all inertial measurements are expected to be zero. Therefore, any recorded non-zero readings are attributed solely to sensor noise, which forms the basis for modeling and characterizing the underlying stochastic error processes. To statistically characterize the inertial measurement time series, normality was first assessed using Q–Q plots and histograms overlaid with fitted Gaussian curves. An ADF test was then applied to determine whether each time series was stationary. Subsequently, the homogeneity of variance across all segmented observations was evaluated using Bartlett’s test, which assumes that the data are approximately normally distributed. Following these preliminary statistical assessments, an ARIMA model was fitted to each time series to identify and characterize its underlying stochastic structure. Finally, the LB test was applied to the model residuals to assess their independence and verify the adequacy of the fitted stochastic model. In addition, the PSD was evaluated, and the slope of the line fitted to its log–log representation was estimated. The results of the analysis demonstrate that the acceleration components along the three orthogonal axes, as well as the angular velocity components around the three orthogonal axes, exhibit purely random behavior. This confirms that the inertial sensor outputs in static mode conform to a white-noise process, implying that the measurement errors are temporally uncorrelated and possess constant variance.

Also, during the 40 min static experiment, sensor temperature was neither actively controlled nor directly measured. Nevertheless, the statistical analyses, including the Allan variance, PSD, and ARIMA-based stochastic modeling, did not reveal substantial bias instability or other low-frequency noise characteristics typically associated with thermally induced sensor drift. This observation suggests that any temperature-related effects were negligible over the duration of the experiment and did not significantly influence the estimated stochastic models. A plausible explanation is that the flagship smartphone used in this study incorporates an efficient thermal management system and power-efficient hardware, resulting in relatively stable operating conditions during static data acquisition. However, this interpretation should not be generalized to all smartphone platforms.

Following the evaluation and preprocessing of the inertial raw data, the measured acceleration and angular velocity signals, representing primarily random noise in the static mode, were subsequently introduced into the strapdown inertial navigation mechanization equations. This procedure enabled the computation of the corresponding attitude, velocity, and position estimates of the smartphone. Under the static experimental assumption, the true attitude, velocity, and position of the device are known to be zero; therefore, any deviations observed in the computed quantities are exclusively attributable to sensor noise and error propagation through the mechanization process. The primary objective of this phase of the study was to investigate the stochastic behavior and noise modeling of the derived attitude, velocity, and position estimates. To this end, the same methodological framework described previously was employed. First, appropriate stochastic models were fitted to characterize the underlying noise processes. The adequacy of the fitted models was then evaluated using the LB test to verify that the residuals exhibited no significant autocorrelation. To enhance the statistical reliability and robustness of the findings, the entire time-series dataset was partitioned into 75 shorter segments (each time series represents 0.5 min of data sampled at 10 Hz), with each segment representing an independent realization of the navigation solution under static conditions. This segmentation allowed for a more comprehensive statistical evaluation of the error characteristics across multiple instances of strapdown integration. The results demonstrate that the velocity and position errors exhibit a random walk behavior, consistent with the cumulative effect of integrating white noise over time. Moreover, the velocity errors show relatively larger variance, reflecting the direct influence of random acceleration noise on velocity estimation. In contrast, the position errors, resulting from the double integration of the same noise source, display a smoother random walk pattern due to the inherent averaging effect of successive integration.

In addition, a sensitivity analysis regarding the window size of the time-series (e.g., 10, 30, and 90 s) was done to show that this methodology is robust to the other window sizes.

Finally, the NNLS-VCE approach was employed to quantitatively assess the stochastic error characteristics of both velocity and position estimates across all 75 segmented time-series obtained from the strapdown mechanization process. By utilizing the NNLS–VCE framework in a multivariate context, it was possible to simultaneously estimate and compare the variance components associated with the different state variables, thereby providing a comprehensive and statistically consistent evaluation of their respective noise levels. This joint analysis enables a more reliable quantification of the precision and relative contribution of each component within the integrated navigation solution.

**Figure 3:**
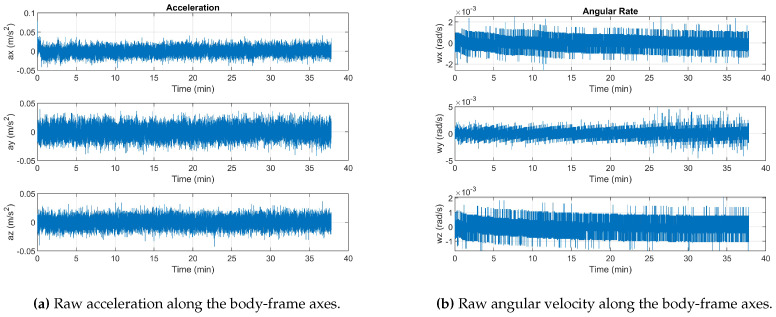
Raw inertial measurements along the body-frame axes.

The raw inertial measurements, comprising acceleration and angular velocity, are shown in Figure 3.

Here, $a_{x}$, $a_{y}$, and $a_{z}$ denote the linear accelerations along the body-frame axes, while $w_{x}$, $w_{y}$, and $w_{z}$ denote the angular velocities along the same axes.

The standard deviation of raw measurements is shown in Table 3.

**Figure 4:**
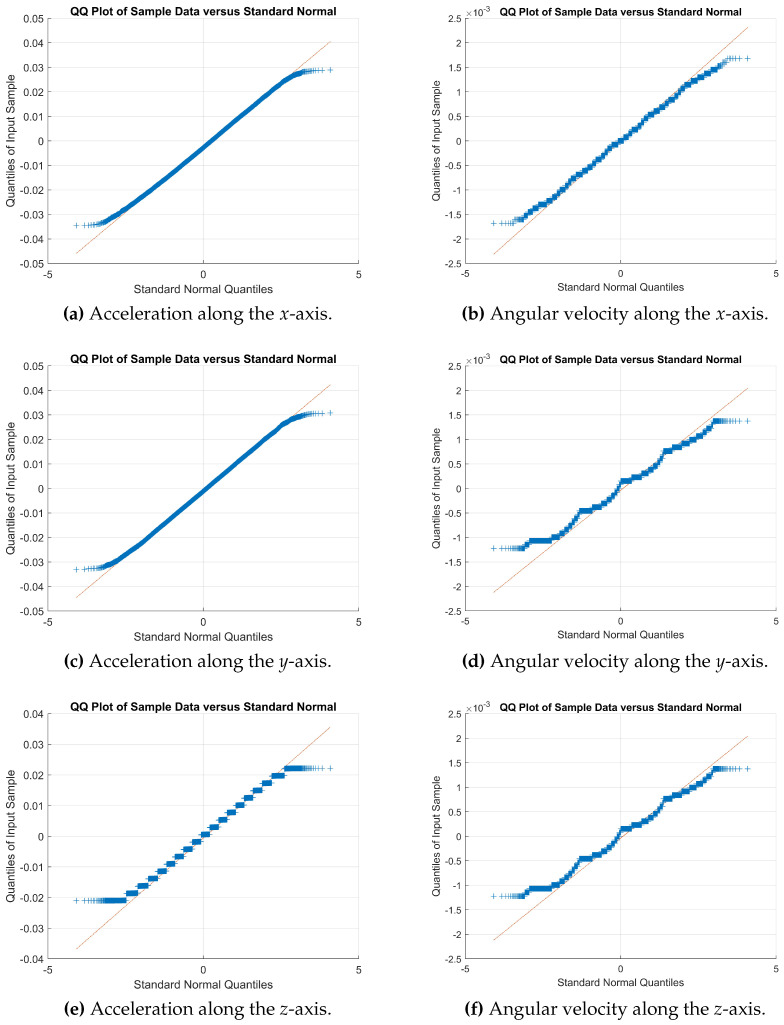
Estimated Q–Q plots of raw accelerometer and gyroscope measurements.

The results of the normality assessment, including the Q–Q plots and the corresponding histograms with fitted Gaussian curves for all inertial measurements, are presented in Figure 4.

**Figure 5:**
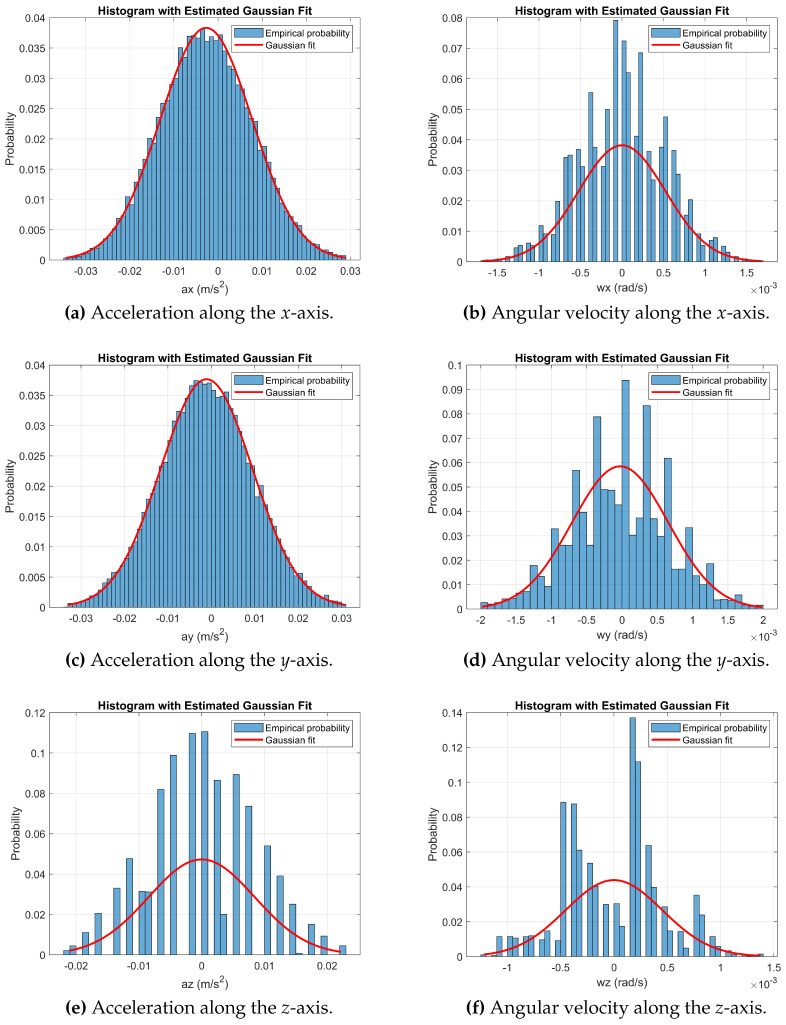
Histograms and estimated Gaussian curves for raw accelerometer and gyroscope measurements.

As shown in these figures, the inertial measurements closely align with the fitted reference line, indicating that the data are well approximated by a Gaussian distribution. Figure 5 also presents the histograms and the corresponding estimated Gaussian curves.

As illustrated in these figures, the probability distributions of the inertial measurements are in good agreement with the fitted Gaussian curves. While some measurements exhibit an excellent fit, the remaining measurements show only minor deviations and can still be regarded as approximately normally distributed.

**Figure 6:**
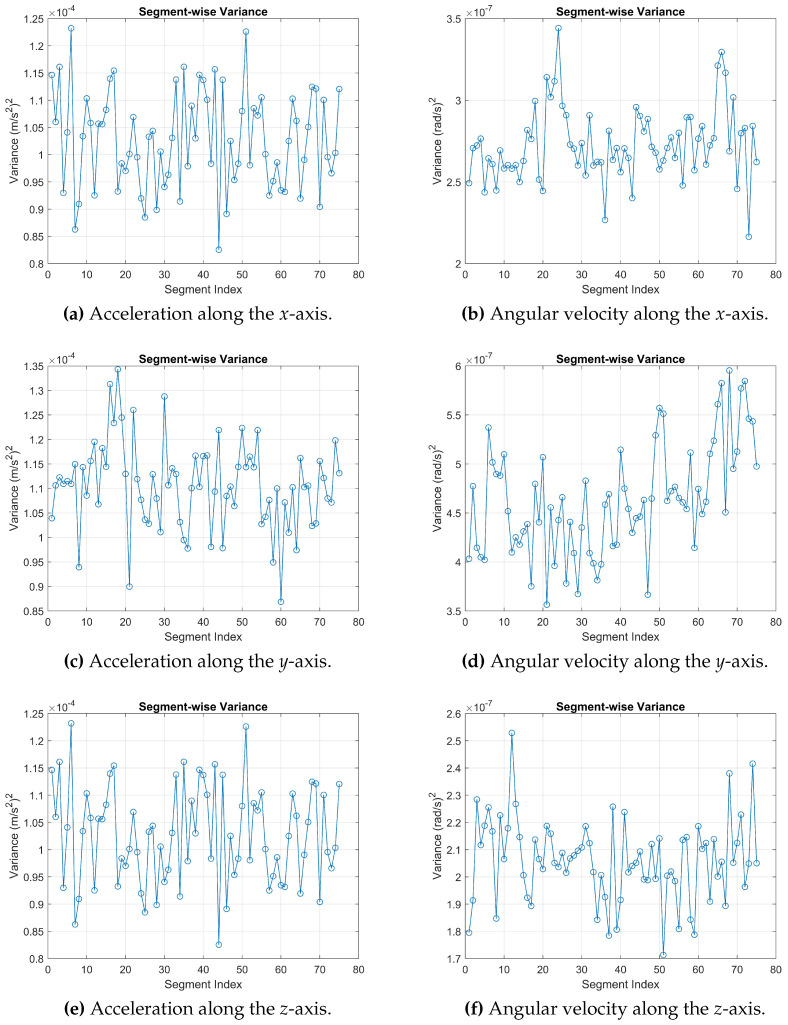
Variation in the variance of raw accelerometer and gyroscope measurements.

Furthermore, Table 4 summarizes the results of Bartlett’s test for homogeneity of variance, whereas Figure 6 illustrates the variance variations among the segmented time series. Together, these results provide a quantitative and visual assessment of the consistency of the variance across all segments.

As shown in Table 4, all $p_{B}$-values obtained from Bartlett’s test are greater than 0.05. Therefore, the null hypothesis of equal variances is not rejected at the 5% significance level, indicating that the segmented time series exhibit statistically homogeneous variances. This result supports the assumption that the segments can be regarded as multiple realizations of the same stochastic process for the subsequent stochastic modeling and variance component estimation.

**Figure 7:**
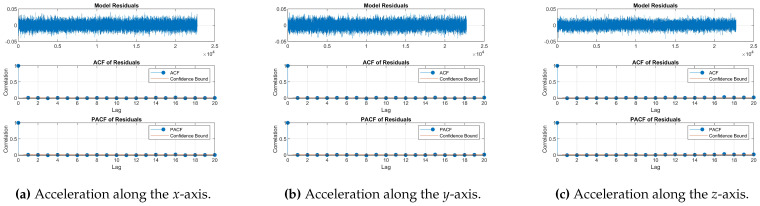
LB test results for the accelerometer components in the body frame.

**Figure 8:**
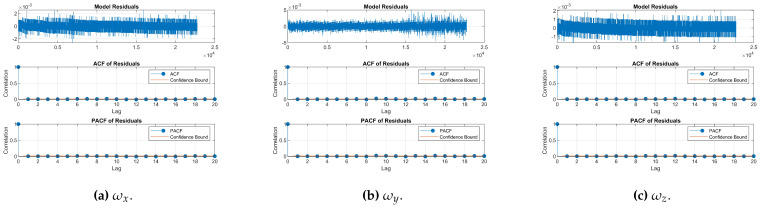
LB test results for the angular velocity components in the body frame.

Based on the results of the statistical tests, as well as the analyses of the ACF and Partial ACF (PACF), the inertial measurements, comprising both acceleration and angular rate components along the three orthogonal axes, can be characterized as white noise. The residuals from the LB test, along with the corresponding ACF and PACF plots for all measurements, are presented in Figure 7 and Figure 8.

**Figure 9:**
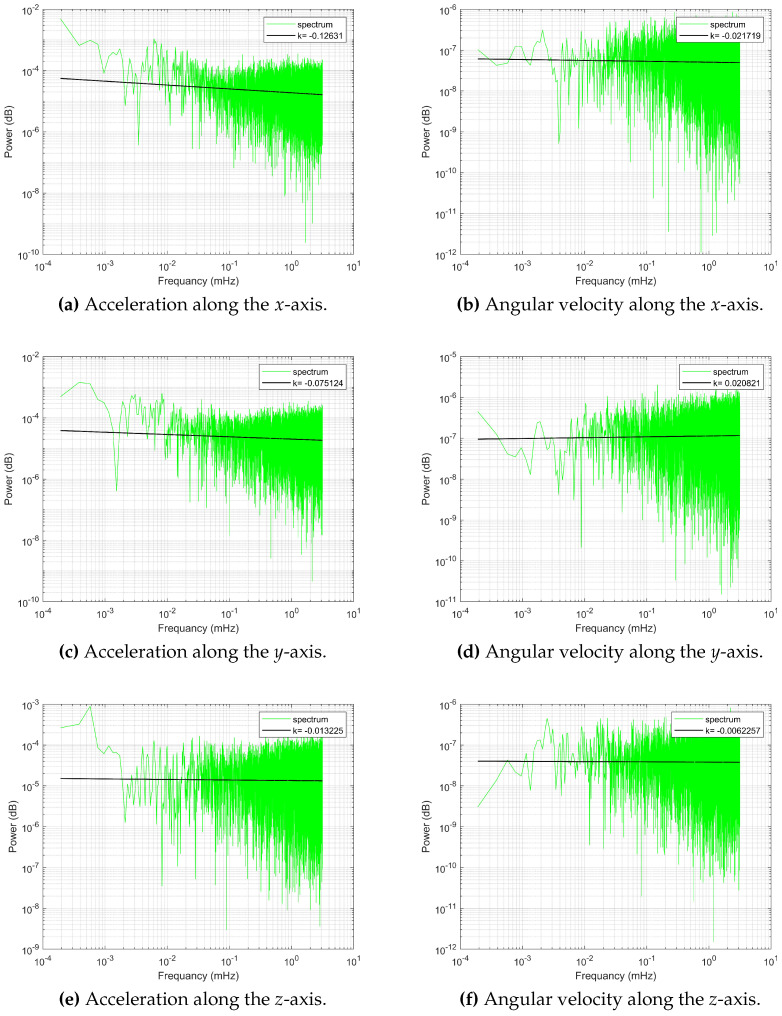
PSDs and estimated spectral slopes of the raw accelerometer and gyroscope signals.

Additionally, Figure 9 shows the computed PSDs and the corresponding slope indices. The slopes are consistently close to κ≈0, indicating that the raw measurements can be modeled as white noise. The slope indices are summarized in Table 5.

The PSD characteristics shown in Figure 9 are limited to approximately 3 mHz because the analysis focuses on the very low-frequency behavior associated with long-term stochastic drift, which is the most relevant frequency range for static MEMS-IMU error characterization. Although the data were sampled at 10 Hz, corresponding to a Nyquist frequency of 5 Hz, the frequency resolution is determined by the total observation time rather than the sampling frequency. Specifically, the complete dataset consists of 40 min (2400 s) of measurements, yielding a frequency resolution of approximately Δν=1/T=1/2400≈0.42 mHz. Consequently, frequencies below a few millihertz are well resolved and provide sufficient information to identify the low-frequency noise characteristics. In contrast, the 30 s segmented time series were used exclusively for the statistical analyses in the time domain (e.g., ADF, ARIMA, and NNLS-VCE) and were not employed for the PSD computation. Therefore, the PSD is presented only up to 3 mHz to emphasize the low-frequency region where long-term stochastic effects are the most evident, while higher-frequency components, although available up to 5 Hz, are less informative for the objectives of this study.

**Figure 10:**
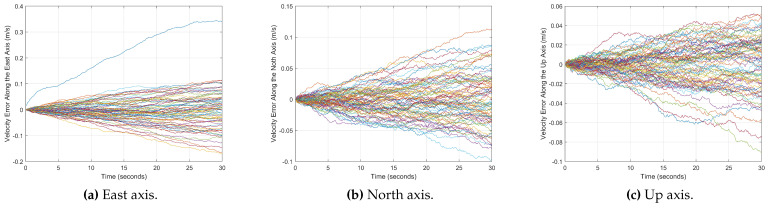
Estimated velocity errors in the ENU frame.

**Figure 11:**
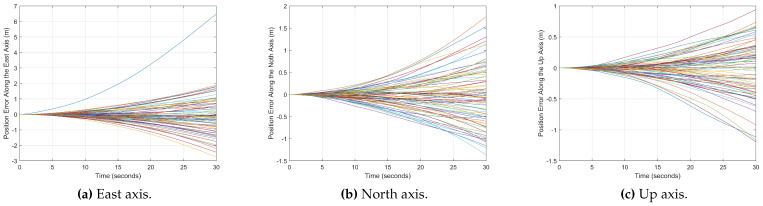
Estimated position errors in the ENU frame.

Subsequently, the entire dataset was segmented into 75 independent time series. For each segment, the strapdown mechanization equations were applied to compute the corresponding velocity and position. The velocity and position estimates for all time series are presented in Figure 10 and Figure 11.

After 30 s of static inertial navigation, the average standard deviations of the ENU velocity estimates are $\sigma_{v}=\left[0.77,0.44,0.29\right]\;m/s$, while the corresponding position standard deviations are $\sigma_{p}=\left[1.30,0.69,0.46\right]\;m$. Although the smartphone remained stationary throughout the experiment, the estimated navigation states exhibit non-zero uncertainties due to the propagation and accumulation of stochastic errors inherent in the MEMS-IMU measurements through the strapdown inertial mechanization. The East component consistently exhibits the largest standard deviation for both velocity and position, whereas the Up component demonstrates the smallest dispersion. This directional variation reflects the combined influence of sensor noise, bias propagation, and the mechanization process, rather than actual platform motion. Furthermore, the larger standard deviations observed for the position estimates compared with the velocity estimates are consistent with the cumulative nature of inertial navigation errors, whereby successive integration amplifies the effects of sensor noise over time. These results quantitatively demonstrate the achievable short-term precision of smartphone-based inertial navigation under static conditions and are in agreement with the stochastic characteristics identified through the ARIMA, PSD, and NNLS-VCE analyses.

**Figure 12:**
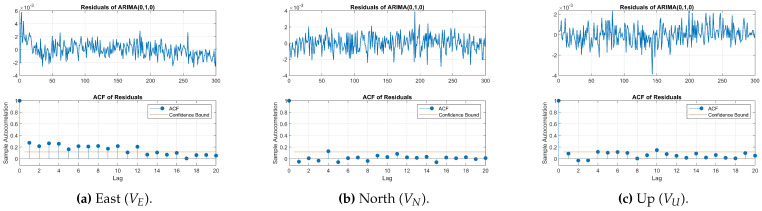
LB test results for the ENU velocity components.

**Figure 13:**
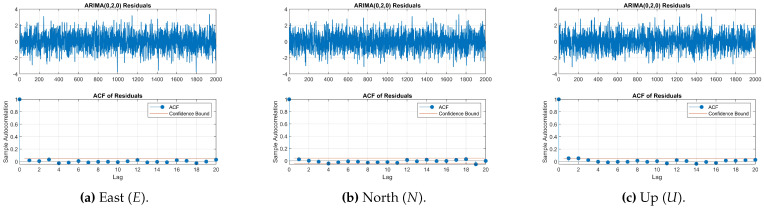
LB test results for the ENU position components.

**Figure 14:**
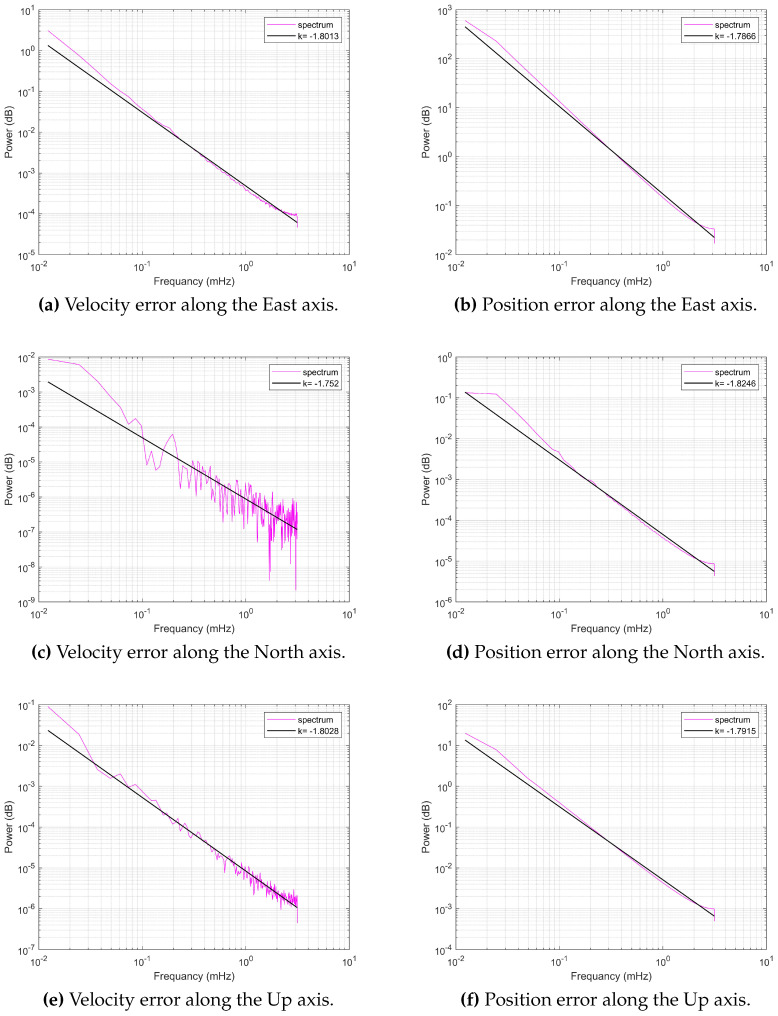
PSDs and the corresponding spectral indices for the estimated velocity and position errors.

Based on the obtained results, the estimated velocity and position errors across all segmented time series exhibit consistent patterns indicative of random walk behavior, or in some cases, a combination of white-noise and random walk characteristics, which is in agreement with the non-stationarity identified by the ADF test. This behavior reflects the presence of stochastic error components that propagate through the strapdown inertial navigation mechanization process. Specifically, the velocity errors are well modeled by an ARIMA(0,1,0) process, indicating that their first differences follow a white-noise sequence and that the errors evolve as a random walk (Figure 12). In contrast, the position errors are adequately represented by an ARIMA(0,2,0) model, implying that their second differences are white noise and corresponding to a second-order integrated random walk process (Figure 13). This distinction is physically consistent with the integration structure of inertial navigation systems, where velocity errors arise from the single integration of white acceleration noise, while position errors result from the double integration of the same noise source. Moreover, the accumulation of these errors over time can be attributed to the inherent amplification of low-frequency noise during the integration of raw acceleration and angular velocity measurements, leading to progressive drift in the estimated states. The PSD analysis further corroborates these findings, with the slopes of the fitted lines being approximately κ≈−2, confirming the random walk nature of both velocity and position errors. The corresponding slope indices are summarized in Table 6, and the results are illustrated in Figure 14.

The slope indices reported in Table 6 provide a quantitative interpretation of the dominant stochastic noise processes present in the inertial measurements and the derived navigation states. Specifically, the slope of the PSD on a log–log scale is directly related to the spectral characteristics of the underlying stochastic process. A slope close to zero indicates white noise, implying that the signal power is approximately uniform across frequencies and successive samples are essentially uncorrelated. In contrast, the velocity and position estimates both exhibit slopes close to −2, indicating the dominance of low-frequency random walk characteristics arising from error accumulation during strapdown inertial mechanization. It should be noted, however, that the PSD slope characterizes the spectral distribution of the signal energy, whereas the ARIMA differencing order quantifies its degree of non-stationarity in the time domain. Consequently, although both velocity and position exhibit similar random walk spectral signatures in the PSD, the ARIMA analysis distinguishes them as ARIMA(0,1,0) and ARIMA(0,2,0) processes, respectively, indicating that the position time series corresponds to an additional level of integration relative to the velocity. Therefore, the PSD slope indices and the ARIMA models provide complementary information, jointly confirming the stochastic behavior and error propagation characteristics of the inertial navigation states.

The PSD value at zero frequency (ν=0) represents the direct-current (DC) component of the signal and is proportional to the square of its mean over the observation interval. Under ideal static conditions, the true velocity and position should have zero mean; therefore, the DC component is expected to be negligible. In practice, however, sensor biases, numerical integration errors, and accumulated drift introduce non-zero mean values, resulting in a finite peak at f=0. Since this component represents the deterministic offset rather than the stochastic noise characteristics of the signal, it is not considered in the interpretation of Figure 14, which focuses on the spectral behavior at non-zero frequencies.

Moreover, the multivariate NNLS-VCE method was applied to all segmented time series to simultaneously estimate the precision associated with each segment. The detailed implementation procedure of this method is provided in Algorithm A1. To address the potential occurrence of negative variance estimates in the conventional formulation, a modified version, referred to as the NN variant, was employed to enforce the non-negativity constraint on the variance components. Based on a preliminary assessment, the covariance structure was modeled as a combination of white noise and random walk components, enabling a comprehensive evaluation of the stochastic behavior and precision characteristics of each individual component. In this algorithm, the covariance matrix is assumed to have the following form:

(53) $$Q=\sigma_{w}^{2}Q_{w}+\sigma_{rw}^{2}Q_{rw},$$

where Q denotes the overall covariance matrix, while $Q_{w}$ and $Q_{rw}$ represent the cofactor matrices corresponding to the white noise and random walk components, respectively. The variance components are $\sigma_{w}^{2}$ and $\sigma_{rw}^{2}$. The white-noise cofactor matrix is modeled as an identity matrix, i.e., $Q_{w}=I$. The random walk cofactor matrix is defined as

(54) $$Q_{rw}=\left[\begin{array}{ccccc} 1 & 1 & 1 & \cdots & 1\\ 1 & 2 & 2 & \cdots & 2\\ 1 & 2 & 3 & \cdots & 3\\ \vdots & \vdots & \vdots & \ddots & \vdots \\ 1 & 2 & 3 & \cdots & m \end{array}\right].$$

Alternatively,

(55) $${\left[Q_{rw}\right]}_{ij}=\min\left(i,j\right),\;i,j=1,2,\ldots ,m.$$

The estimated standard deviations for each navigation component are presented in Table 7. The results indicate that the velocity and position errors exhibit behavior consistent with a pure random walk process (i.e., an integration noise process). This observation is fully consistent with the outcomes of the statistical hypothesis tests and the characteristics revealed by the PSD analysis.

A sensitivity analysis was conducted using window lengths of 10, 30, and 90 s. The estimated white-noise and random walk variance components exhibited only minor variations across the tested window lengths, indicating that the proposed stochastic characterization is robust to the choice of segmentation interval. Consequently, the selected 30 s window provides a suitable compromise between the number of statistical realizations and the reliability of the estimated variance components.

Table 8 presents the sensitivity analysis of the proposed stochastic modeling framework using segmentation window lengths of 10, 30, and 90 s. The estimated white-noise variances of both the accelerometer and gyroscope remain nearly unchanged for all three window lengths, indicating that the white-noise characteristics are essentially independent of the segmentation interval. This observation is expected because the experiments were conducted under static conditions, where the intrinsic sensor noise remains statistically stable throughout the data collection period. Consequently, the proposed methodology provides consistent white-noise estimates regardless of the selected window length.

In contrast, the estimated navigation uncertainties exhibit a clear dependence on the segmentation window length. As the window length increases, the velocity and, more noticeably, the position standard deviations become larger. This behavior results from the cumulative nature of the random walk process, whereby stochastic sensor errors are continuously propagated and integrated through the strapdown inertial navigation equations. Longer data segments therefore permit greater accumulation of these errors, leading to increased navigation uncertainty. Accordingly, the 90 s segmentation produces the largest velocity and position standard deviations, whereas the 10 s segmentation yields the smallest values, with the 30 s window providing intermediate estimates. These findings indicate that the selected 30 s segmentation offers a reasonable compromise between obtaining sufficient statistical realizations for robust stochastic parameter estimation and limiting excessive random walk accumulation within each segment.

Also, the ARW and VRW for acceleration and angular velocity measurements based on Allan variance computation are presented in Table 9.

The estimated VRW and ARW parameters are summarized in Table 9. The VRW coefficients of the three accelerometer axes range from 0.160 to 0.201 $m/s/\sqrt{h}$, while the ARW coefficients of the gyroscope axes range from 0.008 to 0.012 $\mathrm{rad}/\sqrt{h}$. The relatively small variation among the three orthogonal axes indicates that the inertial sensors exhibit consistent stochastic performance without any significant axis-dependent degradation. These values are also consistent with the results obtained from the time-domain analyses, in which the raw accelerometer and gyroscope measurements were identified as stationary white-noise processes. Furthermore, the estimated VRW and ARW coefficients are compatible with the corresponding Allan variance analysis, providing additional validation of the proposed stochastic modeling framework. Since VRW and ARW quantify the accumulation of random measurement errors over time, these parameters provide a practical measure of the achievable velocity and attitude precision of the smartphone MEMS-IMU and serve as representative stochastic characteristics for subsequent inertial navigation error propagation analyses.

The stochastic characteristics obtained from the ARIMA analysis are consistent with the Allan variance results. The Allan deviation analysis identified the dominant accelerometer and gyroscope noise as white noise, corresponding to the VRW and ARW regions, respectively. Since the integration of a white-noise process yields a random walk, the estimated velocity sequence is theoretically expected to follow an ARIMA(0,1,0) model. Furthermore, integrating the random walk velocity produces a second-order integrated process, corresponding to an ARIMA(0,2,0) model for the position sequence. The agreement between the ARIMA-based stochastic models and the Allan variance characterization demonstrates that the inertial navigation errors are primarily governed by white measurement noise over the 40 min observation period. Moreover, the absence of significant autoregressive or moving average components in the identified models is consistent with the inability to reliably identify BI or RRW from the Allan deviation analysis, indicating that these long-term stochastic processes do not dominate the navigation solution within the experimental duration.

## 5. Conclusions and Recommendations

In this study, the stochastic characteristics and precision of a low-cost MEMS-IMU embedded in a Samsung Galaxy S22+ smartphone were systematically investigated under static conditions. Approximately 40 min of inertial measurements was collected while the device remained rigidly fixed at a calibrated zero-reference location in a controlled laboratory environment. Under these conditions, the true specific forces, angular velocities, velocity, and position were assumed to be zero, allowing all non-zero measurements and navigation outputs to be interpreted as stochastic errors. The complete dataset was divided into 75 non-overlapping segments to provide statistically robust estimates and enable multi-realization stochastic analysis.

A comprehensive statistical framework was developed to characterize both the raw inertial measurements and the derived navigation states. The methodology included normality assessment using Q–Q plots and histograms with fitted Gaussian curves, stationarity analysis using the ADF test, variance homogeneity assessment using Bartlett’s test, stochastic model identification using ARIMA models, model validation using the LB test, and complementary frequency-domain characterization using PSD analysis. Furthermore, a multivariate NNLS-VCE method was employed to jointly estimate the variance components of multiple inertial and navigation state variables, providing a unified framework for stochastic error quantification.

Unlike conventional stochastic characterization approaches that primarily focus on raw inertial sensor measurements or employ Allan variance analysis alone, the proposed framework integrates statistical validation, stochastic time-series modeling, multivariate NNLS-VCE, and strapdown inertial navigation error propagation within a unified methodology. This integrated approach enables not only the identification and quantification of stochastic error components in the raw accelerometer and gyroscope measurements but also the direct characterization of the propagated stochastic behavior of the derived velocity and position states. Furthermore, the consistency of the identified stochastic models is independently verified through Allan variance analysis, demonstrating that the proposed framework provides a statistically rigorous and practically applicable methodology for the stochastic modeling and uncertainty quantification of low-cost smartphone MEMS-IMUs.

The results demonstrate that the raw accelerometer and gyroscope measurements are well described by stationary white-noise processes, as confirmed by the statistical tests, PSD slope indices close to zero, and the estimated VRW and ARW coefficients. These findings are also consistent with the corresponding Allan variance analysis. In contrast, the navigation velocity and position estimates exhibit random walk behavior resulting from the accumulation of sensor noise through strapdown inertial mechanization. The velocity states are best represented by an ARIMA(0,1,0) model, whereas the position states follow an ARIMA(0,2,0) model, reflecting the additional integration inherent in position estimation. The PSD analysis further supports these findings by yielding slope indices close to −2 for both velocity and position.

The random walk component serves a role beyond merely characterizing sensor performance. It represents the cumulative propagation of stochastic errors through the strapdown inertial navigation mechanization, whereby successive integrations of white-noise measurements produce non-stationary errors in the derived navigation states. Consequently, the random walk model provides a realistic description of the temporal evolution of velocity and position uncertainties, enabling quantitative precision assessment, covariance modeling, and uncertainty propagation in inertial navigation systems. These stochastic models are particularly valuable for the design of navigation filters, such as Kalman filters and other sensor fusion algorithms, where an accurate covariance structure directly influences state estimation accuracy and filter consistency.

It should be emphasized that the identified ARIMA(0,1,0) and ARIMA(0,2,0) models are valid for the controlled static conditions investigated in this study, where the raw inertial measurements are well characterized as stationary white-noise processes with negligible bias instability. Under these conditions, the models accurately describe the propagation of stochastic errors into the velocity and position estimates. For long-term navigation or environments in which significant sensor biases, colored noise, or temperature-dependent drift are present, more general stochastic models incorporating additional autoregressive or moving average components may be required. Nevertheless, the proposed framework remains practically relevant because it provides a statistically validated baseline methodology for identifying stochastic models directly from experimental data. This methodology can be readily extended to more complex sensor behaviors and offers a systematic foundation for stochastic modeling, covariance estimation, and adaptive filter design in practical inertial navigation and sensor fusion applications.

The NNLS-VCE analysis indicates that the navigation state uncertainties are dominated by random walk components, with negligible white-noise contributions. After 30 s of static navigation, the average standard deviations of the ENU velocity estimates were $\sigma_{v}=\left[0.77,0.44,0.29\right]\;m/s$, while the corresponding position standard deviations were $\sigma_{p}=\left[1.30,0.69,0.46\right]\;m$. These results provide a quantitative assessment of the achievable short-term precision of smartphone-based inertial navigation under static conditions and demonstrate the effectiveness of the proposed statistical framework for stochastic error characterization.

A sensitivity analysis was further conducted using segmentation window lengths of 10, 30, and 90 s to evaluate the robustness of the proposed stochastic modeling framework. The results demonstrated that the estimated white-noise variances of both the accelerometer and gyroscope remained nearly unchanged across all tested window lengths, confirming the stability of the intrinsic sensor noise under static operating conditions. In contrast, the navigation velocity and position uncertainties increased with the window length due to the cumulative nature of the random walk process during inertial error propagation. Considering both the stability of the estimated stochastic parameters and the trade-off between statistical reliability and random walk accumulation, a segmentation window of 30 s is recommended for static stochastic characterization of low-cost smartphone MEMS-IMUs. This window provides sufficient statistical realizations while limiting excessive navigation error growth, thereby offering a balanced choice for reliable stochastic parameter estimation.

Although sensor temperature was not directly monitored during the experiments, no substantial bias instability or other temperature-induced low-frequency effects were observed over the 40 min data collection period. Nevertheless, these results should not be generalized to all smartphone platforms, as devices with different MEMS-IMU architectures, thermal management capabilities, or operating conditions may exhibit more pronounced temperature-dependent behavior.

The proposed methodology is readily applicable to other low-cost MEMS-IMU platforms and can be extended to dynamic navigation scenarios. Future work will focus on controlled kinematic experiments, comparative evaluations across different smartphone models and IMU grades, explicit investigation of temperature effects through controlled thermal experiments, and the incorporation of the identified stochastic models into adaptive sensor fusion algorithms, such as GNSS/INS integration based on Kalman or particle filtering, to further improve navigation performance in GNSS-denied environments.

## Figures and Tables

**Table 1:** Comparison of ARMA and ARIMA models.

Feature	ARMA	ARIMA
Stationarity requirement	Time series must be stationary	Can model non-stationary series after differencing
Components	AR(p) + MA(q)	AR(p) + I(d) + MA(q), where I = differencing order
Differencing	Not included	Includes differencing to achieve stationarity
Use case	Stationary time-series forecasting	Non-stationary or integrated time-series forecasting
Model complexity	Generally simpler	Slightly more complex due to differencing term
Notation	ARMA(p,q)	ARIMA(p,d,q)
Examples	Models stationary noise, temperature anomalies, etc.	Models stock prices, cumulative sums, or trends in sensor data

**Table 2:** Noise model identification from PSD slopes (log–log scale).

Noise Type	Slope (κ)	Comment
White noise	≈0	No temporal correlation
Flicker noise (1/ν)	≈−1	Long-term correlation
Random walk noise	≈−2	Integrated process
Flicker random walk	≈−3	Strong low-frequency drift
Quantization noise	≈+1	High-frequency dominance

**Table 3:** Standard deviation of raw inertial measurement components.

Measurement	Standard Deviation $\sigma_{0}$
$a_{x}$	$0.0106\;m/s^{2}$
$a_{y}$	$0.0107\;m/s^{2}$
$a_{z}$	$0.0088\;m/s^{2}$
$\omega_{x}$	$5.2927\times 10^{-4}\;\mathrm{rad}/s$
$\omega_{y}$	$7.3481\times 10^{-4}\;\mathrm{rad}/s$
$\omega_{z}$	$4.6372\times 10^{-4}\;\mathrm{rad}/s$

**Table 4:** Bartlett’s test $p_{B}$-values for the raw inertial measurements.

Component	$p_{B}$-Value
$a_{x}$	0.18
$a_{y}$	0.51
$a_{z}$	0.93
$\omega_{x}$	0.53
$\omega_{y}$	0.61
$\omega_{z}$	0.89

**Table 5:** Estimated PSD slope indices (κ) for the raw inertial measurements.

Component	κ
$a_{x}$	−0.1263
$a_{y}$	−0.0751
$a_{z}$	−0.0132
$\omega_{x}$	−0.0217
$\omega_{y}$	0.0208
$\omega_{z}$	−0.0062

**Table 6:** Estimated PSD slope indices (κ) for the navigation velocity and position estimates.

Component	κ
*E*	−1.8013
*N*	−1.7520
*U*	−1.8028
$V_{E}$	−1.7866
$V_{N}$	−1.8246
$V_{U}$	−1.7915

**Table 7:** Estimated white-noise and random walk standard deviations of the navigation state components obtained using the NNLS-VCE method.

Component	Unit	$\sigma_{w}$	$\sigma_{\mathrm{rw}}$
*E*	m	0	2.8310
*N*	m	0	2.8284
*U*	m	0	2.8370
$V_{E}$	m/s	0	2.3530
$V_{N}$	m/s	0	2.3717
$V_{U}$	m/s	0	2.3888

**Table 8:** Sensitivity analysis of the estimated white-noise variances and navigation uncertainties for different segmentation window lengths.

Window (s)	White-Noise Variance			Velocity Std. Dev. (m/s)				Position Std. Dev. (m)		
	Acc.	Gyro.		E	N	U		E	N	U
10	0.0083	$5.923\times 10^{-4}$		0.2420	0.0030	0.0070		0.0174	0.0380	0.0440
30	0.0084	$6.249\times 10^{-4}$		0.0710	0.0040	0.0130		1.3388	0.1360	0.2880
90	0.0084	$6.811\times 10^{-4}$		0.1456	0.0561	0.0186		2.6194	1.9888	0.5543

**Table 9:** Estimated velocity random walk (VRW) and angular random walk (ARW) parameters for the accelerometer and gyroscope measurements.

Component	VRW ($m/s/\sqrt{h}$)	ARW ($\mathrm{rad}/\sqrt{h}$)
$a_{x}$	0.197	–
$a_{y}$	0.201	–
$a_{z}$	0.160	–
$\omega_{x}$	–	0.009
$\omega_{y}$	–	0.012
$\omega_{z}$	–	0.008

## Data Availability

The datasets generated and/or analyzed during the current study are available from the first author upon reasonable request. Requests for access to the data should be directed to m.kariminejad@ut.ac.ir.

## Abbreviations

The following abbreviations are used in this manuscript:

ACF	Autocorrelation function
ADF	Augmented Dickey–Fuller
AIC	Alaike information criterion
ARIMA	AutoRegressive integrated moving average
AV	Allan variance
BIC	Bayesian information criterion
BLE	Bluetooth low energy
GMWM	Generalized method of wavelet moments
GNSS	Global navigation satellite systems
IMU	Inertial measurement unit
INS	Inertial navigation system
IoT	Internet of Things
LB	Ljung–Box
MEMS	Microelectromechanical systems
MLE	Maximum likelihood estimation
NNLS-VCE	Non-negative least squares variance component estimation
PSD	Power spectral density
SINS	Strapdown inertial navigation system
UWB	Ultra-wideband.

## Appendix A

The multivariate, non-negative least squares variance component estimation pseudo-code is presented here.

**Algorithm A1** Multivariate NNLS-VCE estimation procedure.
**Require:** Design matrix A; observation vector y; cofactor matrices $Q_{k}$ for k=0,…,m; initial variance vector $\sigma^{\left(0\right)}$; convergence tolerance ϵ **Ensure:** Estimated variance components $\hat{\sigma }$ and covariance matrix $Q_{\hat{\sigma }}$ 1: Detect and remove gross errors in y 2: Set outer iteration index i←0 3: **repeat** 4: Construct covariance matrix: $$Q_{y}=Q_{0}+\sum_{k=1}^{m}\sigma_{k}^{\left(i\right)}Q_{k}$$ 5: Compute normal matrix N and vector l 6: Define NNLS objective: $$\min_{\sigma \geq 0}\left(\frac{1}{2}\sigma^{T}N\sigma -l^{T}\sigma \right)$$ 7: Initialize inner loop: $\sigma^{\left(0\right)}\leftarrow 0$, $\mu^{\left(0\right)}\leftarrow -l$, j←0 8: **repeat** 9: Set $\sigma^{\left(j+1\right)}\leftarrow \sigma^{\left(j\right)}$ 10: **for** k=1 to *m* **do** 11: Update component: $$\sigma_{k}^{\left(j+1\right)}=\max\left(0,\;\sigma_{k}^{\left(j\right)}-\frac{\mu_{k}^{\left(j\right)}}{n_{kk}}\right)$$ 12: Update gradient: $$\mu^{\left(j+1\right)}\leftarrow \mu^{\left(j\right)}+\left(\sigma_{k}^{\left(j+1\right)}-\sigma_{k}^{\left(j\right)}\right)n_{\left(:,k\right)}$$ 13: **end for** 14: j←j+1 15: **until** $∥\sigma^{\left(j\right)}-\sigma^{\left(j-1\right)}∥\;<\epsilon$ 16: Update outer solution: $\sigma^{\left(i+1\right)}\leftarrow \sigma^{\left(j\right)}$ 17: i←i+1 18: **until** $∥\sigma^{\left(i\right)}-\sigma^{\left(i-1\right)}∥\;<\epsilon$ 19: Compute projection matrix: $$P_{C}^{⊥}=I-C{\left(C^{T}N^{-1}C\right)}^{-1}C^{T}N^{-1}$$ 20: Final estimates: $$\hat{\sigma }=\sigma^{\left(i\right)},\;Q_{\hat{\sigma }}=N^{-1}P_{C}^{⊥}$$
